# Modulation of the glycaemic index value of shortbread cookies by the use of erythritol and fruit pomace

**DOI:** 10.1038/s41598-024-65108-y

**Published:** 2024-06-20

**Authors:** Ewa Raczkowska, Maciej Bienkiewicz, Robert Gajda

**Affiliations:** Department of Human Nutrition, Faculty of Biotechnology and Food Science, Wroclaw University of Environmental and Life Sciences, 37 Chelmonskiego Street, 51-630 Wroclaw, Poland

**Keywords:** Diseases, Green chemistry

## Abstract

Fruit pomace, as a by-product of fruit and vegetable processing, is a cheap and easily accessible material for further processing that can replace selected recipe ingredients, most often flour. In addition, their advantage is their high health-promoting potential. The aim of this study was to evaluate the effect of the simultaneous use of erythritol (100% sucrose substitution) and the addition of varying amounts of blackcurrant, chokeberry and apple pomace (0%, 10%, 30% and 50% by weight of flour) on the glycaemic response after consumption of shortbread cookies in an in vivo study with humans (ISO 26642:2010). It was shown that an increase in the addition of each type of pomace reduced the glycaemic index value of the cookies. The pomace and sucrose-sweetened cookies were classified in the medium and low GI group. For each type of pomace, an increase in its share in the recipe of cookies was associated with a reduction in GI values (pomace: apple 49.1–37.2%, blackcurrant 56.4–41.0%, chokeberry 59.4–35.5%). Similar correlations were shown for the use of erythritol (pomace: apple 39.5–29.1%, blackcurrant 43.9–31.9%, chokeberry 34.6–20.7%). A significant effect of pomace addition on the GI values of shortbread cookies, was only observed for sucrose-sweetened products. The results obtained allow the conclusion that there is potential for the use of waste raw materials in the production of functional foods.

## Introduction

The glycaemic index (GI) value of foods/meals has a key role in the management of diabetes. Studies indicate that a low GI diet can positively affect postprandial glucose metabolism, tissue insulin sensitivity and glycaemic control in people with carbohydrate metabolism disorders^1,2^. The implementation of a low-GI diet can lead to a reduction in glucose exposure, insulin secretion and total insulin clearance, which helps to control blood glucose concentration^3^. The above observations are also supported by a meta-analysis of the results of 29 studies involving 1617 patients diagnosed with type 1 and type 2 diabetes, which showed that a diet based on low GI foods resulted in significant improvements in patients' health, including glycaemic control, blood lipid levels, weight reduction, normalisation of blood pressure and alleviation of inflammation in the body. Such a multifaceted health impact minimises the risk of diabetes complications^4^.

The use of fruit pomace is an alternative for the food industry. Fruit peels are used, among other things, to isolate pectin, a soluble fibre with proven positive effects on the body^5,6^. The pomace can retain nutritional and bioactive compounds such as phenolic acids, flavonoids and anthocyanins, among others, with different biological activity^7^. Thus, it is a valuable source of bio-compounds that can find application in the production of functional food additives. Less important for the consumer, but crucial for the industry, is the management of waste, thus minimizing its negative impact on the environment^8^. The study results further suggest that the inclusion of fruit pomace in foods may be a valuable strategy to modulate glycaemic responses and improve the nutritional profile of foods. This is demonstrated in the studies of Guzman et al*. *^9^, Alongi et al*.*^10^, Chen et al*.*^11^, Zuñiga-Martínez et al*.*^12^, among others.

Changing consumer habits and lifestyles, over the past half century, have also brought a significant change in the way we consume and use sugar. In addition, the development of low-sugar foods among people with diabetes, as well as among diet-conscious people and the growing demand for diet foods, is expected to accelerate the growth of the market for sugar substitutes. Therefore, many ingredients currently used as sweeteners contain sugar alcohols and other organic and synthetic sweeteners in addition to sucrose^13^. One such substitute is erythritol, which exhibits a number of health properties, including beneficial effects on oral health and hypoglycaemia, and anti-hyperglycaemia, anti-diabetic and anti-obesity effects^14,15^. Furthermore, studies indicate that erythritol may lower GI by reducing glucose absorption in the small intestine, increasing glucose uptake by muscles and improving the activity of glucose-metabolising enzymes^16^. In addition, the use of erythritol in the production of sweet snack products is supported by its functional and technological properties, such as its stability at temperatures above 160 °C and the absence of side effects even with daily consumption. As a result, it is currently the most widely used substitute for sucrose in the production of low-calorie confectionery^17^.

In the available literature, research results on the effect of the addition of fruit pomace or sucrose substitutes to food products most often on their nutritional value, antioxidant properties and organoleptic characteristics can be found^10,18–20^. To our knowledge, there are few research results determining the effect of such waste materials on the glycaemic response after consumption of food products with their addition. A study by Umbreen et al*.* assessed the effect of the addition of apple pomace and mango peel powder to biscuits on postprandial glucose and insulin concentrations. It was shown that their addition at a level of 15% contributed to a significant reduction in the indicated blood biochemical parameters^21^. Similar observations were made in a study by Alongi et al*.*, where in shortcrust pastries wheat flour was partially replaced with apple pomace (10 and 20%), the glycaemic index of the products decreased from 70 to 65% and 60%, respectively^10^. The innovative element of the present study is to evaluate the effect of the simultaneous use of fruit pomace and erythritol on the glycaemic response after the consumption of shortbread cookies. Additionally, in the studies conducted to date, the addition of pomace most often ranged from 8 to 30%^10,18–20^. Considering the beneficial effects of both erythritol and fruit pomace on the GI reduction of products and the lack of results from studies testing their synergistic effects, an attempt was made to assess the effect of the simultaneous use of erythritol and the addition of varying amounts of blackcurrant, chokeberry and apple pomace (0%, 10%, 30% and 50% by weight of flour) on the glycaemic response after consumption of shortbread cookies in an in vivo study.

## Materials and Methods

### Composition and preparation of tested products

Fruit pomace has been purchased from a certified company GreenHerb-Kuźniar Dariusz (Łańcut, Poland). GreenHerb-Kuźniar Dariusz receives wet pomace from fruit juice producers. The pomace is then dried in the Agromech M829 tumble dryer at 70 °C for 3 h. After drying, the pomace is crushed as needed on Scorpion slicers and grinders and sieved using sifters from the same manufacturer. The other ingredients (wheat flour type 450, sucrose, erythritol, butter, eggs) necessary to make the cookies were purchased from retail outlets (Wroclaw, Poland).

Each shortbread cookie variant consisted of wheat flour (type 450), sucrose/erythritol, fruit pomace, butter (82% fat) and egg yolks. The proportions of sucrose/erythritol, butter and egg yolks were the same in each variant and were respectively: 15.2%, 30.3% and 9.1%. The proportion of wheat flour and fruit pomace varied according to the cookie variant (Table 1). The following parameters were used during the production of the shortbread cookies: mixing of dry ingredients (wheat flour, sucrose/erythritol and fruit pomace) [t = 2 min, KitchenAid mixer model 5KPM5 (Springfield, OH, USA)]; dough kneading [t = 3 min, KitchenAid mixer model 5KPM5 (Springfield, OH, USA)]; cooling of the dough [t = 60 min; T = 4 °C]; rolling out the dough [thickness—0.5 cm]; cookie-cutting [diameter—5 cm], baking [t = 8 min; T = 180 °C, Rational Combi Steamer convection-steam furnace (Landsberg am Lech, Munich, Germany)]; conditioning [t = 30 min; T = 22 °C].

**Table 1:** Composition of the different shortbread cookie variants.

	Wheat flour (type 450)	Sucrose	Erythritol	Butter (82% fat)	Egg yolk	Apple/chokeberry/blackcurrant pomace
SCC1; SCC2; SCC3	600.0 g (45.4%)	200.0 g (15.2%)	0.0 g (0.0%)	400.0 g (30.3%)	120.0 g (9.1%)	0.0 g (0.0%)
SA10; SC10; SB10	540.0 g (40.9%)	200.0 g (15.2%)	0.0 g (0.0%)	400.0 g (30.3%)	120.0 g (9.1%)	60.0 g (4.5%)
SA30; SC30; SB30	420.0 g (31.8%)	200.0 g (15.2%)	0.0 g (0.0%)	400.0 g (30.3%)	120.0 g (9.1%)	180.0 g (13.6%)
SA50; SC50; SB50	300.0 g (22.7%)	200.0 g (15.2%)	0.0 g (0.0%)	400.0 g (30.3%)	120.0 g (9.1%)	300.0 g (22.7%)
ECC1; ECC2; ECC3	600.0 g (45.4%)	0.0 g (0.0%)	200.0 g (15.2%)	400.0 g (30.3%)	120.0 g (9.1%)	0.0 g (0.0%)
EA10; EC10; EB10	540.0 g (40.9%)	0.0 g (0.0%)	200.0 g (15.2%)	400.0 g (30.3%)	120.0 g (9.1%)	60.0 g (4.5%)
EA30; EC30; EB30	420.0 g (31.8%)	0.0 g (0.0%)	200.0 g (15.2%)	400.0 g (30.3%)	120.0 g (9.1%)	180.0 g (13.6%)
EA50; EC50; EB50	300.0 g (22.7%)	0.0 g (0.0%)	200.0 g (15.2%)	400.0 g (30.3%)	120.0 g (9.1%)	300.0 g (22.7%)

S—sucrose; E—erythritol; A—apple pomace; C—chokeberry pomace; B—blackcurrant pomace; 10, 30, 50—% addition of pomace to flour weight; SCC 1, 2, 3—control cookies sweetened with sucrose; ECC 1, 2, 3—control cookies sweetened with erythritol.

### Nutritional value of shortbread cookies

The energy and nutritional value of the tested cookies were determined using standard methods. The energy value was determined using the Rosenthal method^22^. The methods of the Association of Official Analytical Chemists (AOAC) were used for other determinations: dry matter (AOAC 925.49-1925), ash (AOAC 940.26), proteins (AOAC 920.152), dietary fibre (AOAC 985.29) and fat (AOAC 996.06)^23^. The amount of available carbohydrates in the tested shortbread cookies was calculated according to the following formulae:

- for sucrose-sweetened shortbread cookies
  available carbohydrates = dry matter − (dietary fibre + fat + protein + ash).
- for erythritol-sweetened shortbread cookies
  available carbohydrates = dry matter − (dietary fibre + fat + protein + ash + erythritol).

The portion size consumed by the study participants depended on both the proportion of pomace in the recipe and the type of sweetener used. As erythritol as a sugar alcohol is not included in the pool of digestible carbohydrates, the portions of cookies consumed were larger than those of sucrose-sweetened products. This translates simultaneously into their higher energy value and macronutrient content.

Nutritional value of a serving of each shortbread cookie variants, containing 25 g of assimilable carbohydrates is shown in Table 2.

**Table 2:** Nutritional value of a serving of each shortbread cookie variants containing 25 g of assimilable carbohydrates.

Variant of shortbread cookies	Portion (g)	Ash (g)	Dry matter (g)	Energy value (kcal)	Protein (g)	Fat (g)	Carbohydrates* (g)	Dietary fiber (g)	Assimilable carbohydrates (g)
		$\overline{x} \pm {\text{SD}}$	$\overline{x} \pm {\text{SD}}$	$\overline{x} \pm {\text{SD}}$	$\overline{x} \pm {\text{SD}}$	$\overline{x} \pm {\text{SD}}$	$\overline{x} \pm {\text{SD}}$	$\overline{x} \pm {\text{SD}}$	$\overline{x} \pm {\text{SD}}$
SCC1	46.0	0.203 ± 0.01	44.60 ± 0.01	237.5 ± 2.34	3.84 ± 0.03	14.20 ± 0.03	26.28 ± 0.02	1.28 ± 0.06	25.00 ± 0.04
SA10	49.0	0.253 ± 0.00	47.74 ± 0.02	244.5 ± 2.89	3.69 ± 0.12	15.46 ± 0.14	28.34 ± 0.29	3.35 ± 0.03	24.99 ± 0.26
SA30	60.0	0.380 ± 0.00	58.32 ± 0.01	296.0 ± 1.13	4.36 ± 0.07	19.41 ± 0.05	34.16 ± 0.14	9.17 ± 0.16	25.00 ± 0.02
SA50	72.0	0.525 ± 0.01	69.78 ± 0.01	349.7 ± 7.22	4.89 ± 0.17	23.17 ± 0.10	41.19 ± 0.29	16.18 ± 0.22	25.00 ± 0.51
ECC1	65.0	0.289 ± 0.01	60.92 ± 0.02	277.9 ± 7.42	4.44 ± 0.16	19.24 ± 1.09	36.95 ± 0.96	2.07 ± 0.02	25.00 ± 0.98
EA10	75.0	0.363 ± 0.02	70.02 ± 0.03	311.5 ± 6.12	5.11 ± 0.18	22.86 ± 0.07	41.66 ± 0.08	5.28 ± 0.03	25.00 ± 0.12
EA30	94.0	0.572 ± 0.01	90.14 ± 0.03	380.7 ± 5.24	6.39 ± 0.22	29.54 ± 0.08	53.65 ± 0.12	14.44 ± 0.21	25.00 ± 0.33
EA50	126.0	0.884 ± 0.04	120.4 ± 0.01	486.1 ± 13.47	6.63 ± 0.00	40.13 ± 0.45	72.73 ± 0.49	28.64 ± 0.02	25.00 ± 0.47
SCC2	46.0	0.203 ± 0.01	44.56 ± 0.01	237.5 ± 2.34	3.84 ± 0.03	14.24 ± 0.03	26.28 ± 0.02	1.28 ± 0.06	25.00 ± 0.04
SC10	50.0	0.259 ± 0.00	48.74 ± 0.04	243.1 ± 1.65	3.79 ± 0.17	15.68 ± 0.05	29.03 ± 0.16	4.03 ± 0.10	25.00 ± 0.26
SC30	63.0	0.420 ± 0.00	61.47 ± 0.01	302.3 ± 0.73	4.38 ± 0.00	20.36 ± 0.11	36.32 ± 0.13	11.32 ± 0.35	25.00 ± 0.48
SC50	81.0	0.634 ± 0.00	79.66 ± 0.01	388.3 ± 12.48	5.10 ± 0.19	26.04 ± 0.04	47.90 ± 0.23	22.90 ± 0.27	25.00 ± 0.05
ECC2	65.0	0.289 ± 0.01	60.92 ± 0.02	277.9 ± 7.41	4.44 ± 0.16	19.24 ± 1.09	36.95 ± 0.96	2.07 ± 0.02	25.00 ± 0.98
EC10	76.0	0.391 ± 0.01	72.62 ± 0.01	308.7 ± 3.06	5.15 ± 0.18	23.91 ± 0.04	43.18 ± 0.24	6.73 ± 0.00	25.00 ± 0.25
EC30	100.0	0.674 ± 0.01	96.91 ± 0.08	393.0 ± 8.84	4.66 ± 0.37	32.27 ± 0.14	59.28 ± 0.14	19.05 ± 0.42	25.00 ± 0.56
EC50	201.0	1.547 ± 0.01	191.8 ± 0.02	767.3 ± 23.4	11.74 ± 0.74	63.61 ± 0.05	114.9 ± 0.64	59.53 ± 0.06	25.00 ± 0.58
SCC3	46.0	0.203 ± 0.01	44.56 ± 0.01	237.5 ± 2.34	3.84 ± 0.33	14.24 ± 0.03	26.28 ± 0.02	1.28 ± 0.06	25.00 ± 0.04
SB10	52.0	0.341 ± 0.00	50.22 ± 0.04	262.5 ± 3.12	4.29 ± 0.06	16.56 ± 0.08	29.00 ± 0.02	3.99 ± 0.21	25.01 ± 0.19
SB30	66.0	0.714 ± 0.01	63.19 ± 0.04	324.5 ± 9.63	5.15 ± 0.00	20.96 ± 0.05	36.35 ± 0.00	11.35 ± 0.14	25.01 ± 0.15
SB50	90.0	1.318 ± 0.03	85.76 ± 1.38	436.9 ± 10.57	7.88 ± 0.00	27.56 ± 0.24	48.21 ± 0.09	23.21 ± 0.51	25.00 ± 0.42
ECC3	65.0	0.289 ± 0.01	60.92 ± 0.02	277.9 ± 7.41	4.44 ± 0.16	19.24 ± 1.09	36.95 ± 0.96	2.07 ± 0.02	25.00 ± 0.98
EB10	75.0	0.478 ± 0.01	70.46 ± 0.06	311.3 ± 2.95	4.85 ± 0.18	23.09 ± 0.10	42.08 ± 0.29	5.70 ± 0.18	25.01 ± 0.10
EB30	110.0	1.153 ± 0.01	104.0 ± 0.01	440.1 ± 3.94	7.67 ± 0.00	33.97 ± 0.01	61.18 ± 0.05	19.53 ± 0.25	25.00 ± 0.21
EB50	243.0	3.658 ± 0.10	236.2 ± 0.09	962.0 ± 3.54	20.77 ± 0.62	81.82 ± 0.47	129.90 ± 0.12	68.01 ± 0.78	25.00 ± 0.66

S—sucrose; E—erythritol; A—apple pomace; C—chokeberry pomace; B—blackcurrant pomace; 10, 30, 50—% addition of pomace to flour weight; SCC 1, 2, 3—control cookies sweetened with sucrose; ECC 1, 2, 3—control cookies sweetened with erythritol.

$\overline{x}$—mean value; SD—standard deviation; *the carbohydrate content also includes the proportion of sucrose/erythritol at 15.2% in each variant of the shortbread cookies.

### Characteristics of the study group

**Figure 1:**
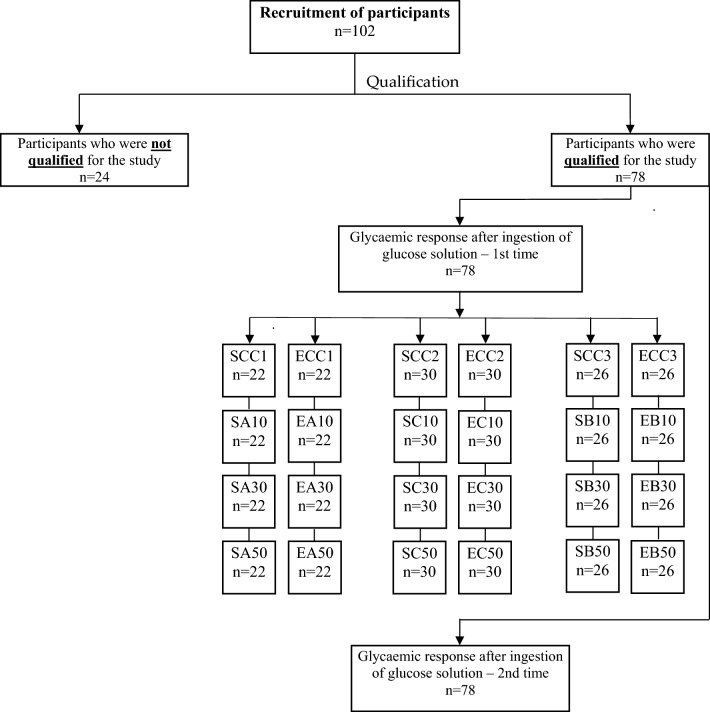
The scheme of the recruitment procedure. S—sucrose; E—erythritol; C—chokeberry pomace; B—blackcurrant pomace; A—apple pomace; 10, 30, 50—% addition of pomace to flour weight; SCC 1, 2, 3—control cookies sweetened with sucrose; ECC 1, 2, 3—control cookies sweetened with erythritol.

The research was conducted with the participation of students from the Wroclaw University of Environmental and Life Sciences in Wroclaw (Poland). The research was conducted between October 2022 and December 2023. Potential participants were presented with the purpose of the study and the next steps in detail. All had the opportunity to ask questions. Participants were additionally informed about the possibility of resigning from participation in the research at any stage of the research, without giving a reason. A total of 102 people were preliminarily qualified for the study and verified against the criteria necessary for the determination of glycaemic index (GI) values^24,25^. To this end, each person completed a questionnaire on their health status, medications used, physical activity, use of stimulants and dietary habits. In addition, anthropometric measurements—height and weight—were taken for each potential participant and the body mass index (BMI) was calculated on this basis. The detailed protocol of the procedure and the final number of participants consuming a specific variant of cookies is shown in Fig. 1. In the end, 78 individuals with a mean age of 21.7 ± 1.2 years and BMI = 21.2 ± 2.1 kg/m^2^ participated in the study. Those qualified for the study met the following criteria:

- age 18–40
- BMI: 18.5–24.9 kg/m^2^
- fasting blood glucose level ≤ 99.0 mg/dL
- no diagnosed diseases and no use of medications that may affect carbohydrate metabolism
- use of usual diet (no special diets)
- physical activity of moderate intensity
- no use of stimulants such as tobacco products or psychoactive substances
- giving written informed consent to participate in the study and maintaining a 10-h fast each time before the study (accessing the study on an empty stomach).

The study was authorized by the Bioethics Committee of the Medical University of Wroclaw, no. KB-848/2021. The study was based on the guidelines of the Declaration of Helsinki^26^.

### Glycaemic response and glycaemic index in an in vivo study

Blood glucose concentrations were measured after drinking a reference solution (25 g of crystalline glucose dissolved in 250 mL of warm boiled water) twice (at the beginning and end of the study) and once after consuming the analysed shortbread cookie variants, sweetened with sucrose and erythritol. Each cookie variant contained 25 g of assimilable carbohydrate (Table 2). The test was performed according to the Report of a Joint FAO/WHO Expert Consultation and ISO 26,642:2010^24,25^. The blood glucose concentration was measured from the fingertip (fasting) using an automatic Accu-Chek Softclix pricker (Roche Diagnostics, Rotkreuz, Switzerland). After each blood drop was collected, blood glucose concentrations were measured using an Accu-Chek Active glucometer (Roche Diagnostics). Qualified study participants were divided into three groups. Each group consumed shortbread cookies with a particular type of pomace, sweetened with sucrose/erythritol. Each variant was consumed by a minimum of 22 people (according to the GI determination methodology, the minimum number of people is 10). A schematic of the experiment is shown in Fig. 1.

The GI of each product variant was the arithmetic mean of the GI values calculated individually for each participant. When the GI value for any participant was higher than the mean ± 2 × standard deviation, this result was considered an outlier and was excluded from the calculation of the mean GI value of the cookie variant. The personal data of the participants were coded in accordance with the guidelines of the General Regulation of the European Parliament on the Protection of Personal Data (GDPR 679/2016).

### Statistical analysis

Results were statistically analysed using Statistica 13.3 PL (StatSoft, Tulsa, OK, USA). The normality of the distribution of the variables was checked using the Shapiro–Wilk test. Certain variables deviated from the normal distribution, so for non-normally distributed data, the Box–Cox transformation was performed to normalise the data. Three-way ANOVA was performed to calculate the p-value of sample interaction between fruit pomace type, % of fruit pomace, and sweetener according to the glycemic index result. Analysis of variance (ANOVA) was performed with significance level set to p < 0.05. The coefficient of variation (CV) values of the glycaemic response after double ingestion of a standard glucose solution did not exceed 18% (the required value is CV < 30%). Data were reported as mean ± standard deviation.

## Results and discussion

### Glycaemic index values of shortbread cookies with chokeberry, apple and blackcurrant pomace

**Figure 2:**
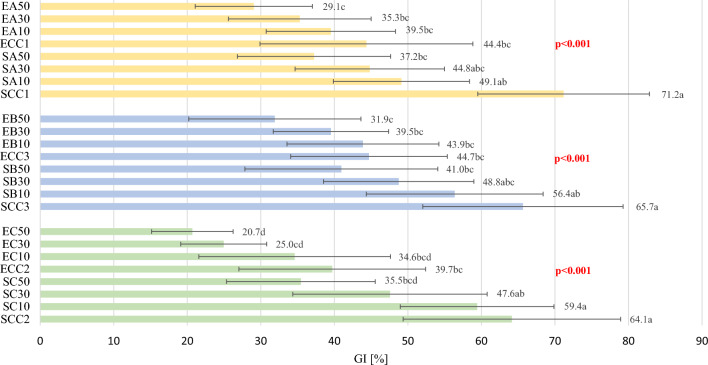
Glycaemic index (GI) values of tested shortbread cookie variants. S—sucrose; E—erythritol; C—chokeberry pomace; B—blackcurrant pomace; A—apple pomace; 10, 30, 50—% addition of pomace to flour weight; SCC 1, 2, 3—control cookies sweetened with sucrose; ECC 1, 2, 3—control cookies sweetened with erythritol; GI—glyceamic index; a, b, …—statistically significant differences in glycaemic index after consumption of shortbread cookies with different proportions of chokeberry/blackcurrant/apple pomace and addition of sucrose or erythritol; *p*—impact of adding chokeberry/blackcurrant/apple and sucrose/erythritol on the glycaemic index of shortbread cookies.

Figure 2 shows the glycaemic index values of the different shortbread cookie variants. In addition, a three-way ANOVA was conducted to determine the effect of pomace type, level of addition and type of sweetener, as well as the interaction between these factors, on the GI values of shortbread cookies. The results are shown in Table 3. Significant correlations were only shown for the main effects: % of fruit pomace (p < 0.001), type of sweetener (p < 0.001) and, at the limit of statistical significance, type of fruit pomace (p = 0.0553). There were no significant two-way or three-way interactions caused by type of pomace, its percentage addition and type of sweetener on the GI values of the shortbread cookies. As can be seen in Table 1, the recipe of SCC1, SCC2, SCC3 and ECC1, ECC2, ECC3 cookies was the same. However, different GI values were obtained for the control shortbread cookies. This is due to the fact that each shortbread cookie variant, depending on the pomace type, was tested by one of three groups of participants (Fig. 1). For each type of pomace, it was shown that the GI value decreased as the proportion of pomace increased. Similar relationships were shown when sucrose was replaced by erythritol, however, statistically significant differences in the GI values of erythritol-sweetened cookies were only shown for shortbread cookies with chokeberry pomace (ECC2 vs. EC50). Regardless of pomace type, products sweetened with sucrose (addition of 0% and 10% of fruit pomace) were classified as products with medium GI (chokeberry, blackcurrant) or high GI (apple). In the case of cookies sweetened with sucrose with apple pomace, the addition of 10% pomace reduced the GI value from high to low (SCC1 vs. SA10), while a statistically significant difference in GI value was observed with 50% addition compared to the SCC1 variant (SCC1 vs. SA50). In the case of both cookies made with chokeberry pomace and those made with blackcurrant pomace, only 30% addition resulted in a reduction of the GI value from medium to low. In contrast, as in the case of apple pomace, addition at the 50% level was statistically significant in relation to the GI values of SCC3 and SCC2.

**Table 3:** Three-way ANOVA showing interaction among fruit pomace type, % of fruit pomace addition, and sweetener observed from glycaemic index (GI) values. Fruit pomace—apple/chokeberry/blackcurrant pomace; % of fruit pomace—0, 10, 30, 50% of fruit pomace addition; Sweetener—sucrose/erythritol.

	Type III sum of squares	Degree of freedom	Mean square	F value	p-value
Fruit pomace	1147.1	2	723.6	2.927	0.0553
% of fruit pomace	20,441.8	3	6813.9	27.560	< 0.001
Sweetener	18,389.7	1	18,389.7	74.379	< 0.001
Fruit pomace*% of fruit pomace	841.5	6	140.3	0.567	0.7562
Fruit pomace* Sweetener	1086.6	2	543.3	2.197	0.1131
% of fruit pomace* Sweetener	1715.2	3	571.7	2.312	0.0765
Fruit pomace*% of fruit pomace* Sweetener	655.9	6	109.3	0.442	0.8502

GI values of cookies made with apple pomace were also determined by Alongi et al*.*^10^. The authors used pomace addition at levels of only 10% and 20% by weight of flour. Products without the addition of pomace were classified as having a high GI (GI = 70.0%), while cookies with 10% and 20% addition achieved GI values of 65.0% and 60.0%, respectively. Within the framework of our study, a similar GI value was obtained for traditional cookies—71.2%, while the GI of the products with 10% addition was 15.9% lower (Fig. 2). The differences are most likely due to different methods of GI determination. This is because Alongi et al. used an in vitro digestion method^10^. In addition, the dietary fibre content of apple pomace varies from 4.4 to 47.3%^27,28^. This component, in fact, has a key effect on blood glucose concentrations after the consumption of carbohydrate products^6^. The by-products of berries, including chokeberries and blackcurrants, in addition to their significant dietary fibre content, are characterized by a high content of polyphenolic compounds. They have been shown to have an inhibitory effect on the activity of α-amylase and α-glucosidase^29^. Inhibition of enzyme activity has been attributed to interactions between enzymes and polyphenolic compounds^30,31^. The health-promoting value of pomace depends, among other things, on the type of fruit and the drying method used^32,33^. In our study, we showed that the addition of both chokeberry and blackcurrant pomace reduced the GI value of shortbread cookies (Fig. 2).

In all cookie variants in which sucrose was replaced by erythritol, it was observed that the replacement of only the sweetener reduced the GI of tested cookies, classifying them as low GI products (20.7–44.7%). Furthermore, statistical analysis showed that only in the case of products with added chokeberry pomace, the difference in GI values was statistically significantly dependent on the addition of pomace (ECC2 = 39.7% vs EC50 = 20.7%) (p = 0.0032). Analysing the data for products with added erythritol and blackcurrant and apple pomace, it was observed that the addition of pomace had no significant effect on GI values (p = 0.0845 and p = 0.2271, respectively). It can therefore be presumed that, when using erythritol, the addition of pomace has a smaller effect on GI values than when using sucrose. Research involving the use of erythritol as a sweetener for bakery products was also carried out by Abo-Zaid. In addition to substituting sucrose with erythritol (100%), the author also used different percentages of oat flour (50%, 75% and 100%). All modified products had a lower GI compared to the control product (the difference ranged from 8.1 to 11.6%)^16^. In this study, the difference between the sucrose- and erythritol-sweetened cookie variants was significantly higher, ranging from 21.0% (SCC3 vs ECC3 ) to 26.8% (SCC1 vs ECC1 ) (Fig. 2). Daily consumption of 20 g of erythritol has been shown to contribute to lower glycated haemoglobin concentrations. At the same time, erythritol has been shown to delay gastric emptying and glucose absorption from the small intestine, as observed by increases in the intestinal hormones glucagon-like peptide 1 (GLP-1), cholecystokinin (CCK) and peptide YY (PYY)^34^. An interesting observation was made by Wen et al*.* showing that erythritol can only impede intestinal glucose absorption when consumed together with a carbohydrate product^35^.

In conclusion, it has been shown that the GI values of shortbread cookies are influenced by both the proportion of fruit pomace in their recipe and the type of sweetener used. However, this effect is dependent on the type of pomace. Considering the classification of food products, according to their GI value^25^, in the case of apple pomace, its addition to sucrose-sweetened cookies as low as 10% resulted in a low GI product. By contrast, in the case of blackcurrant and chokeberry pomace, an addition of 30% is required. By simply replacing sucrose with erythritol, all cookie variants (irrespective of pomace %) can be classified as low GI products. Taking into account consumer expectations of functional foods, it should be emphasised that, in addition to the GI value of the products, their nutritional value, health-promoting properties and sensory characteristics, similar to traditional products are of equal importance^36^. Based on the obtained results, for healthy people choosing sweet snack products with functional characteristics, it is recommended to choose sucrose-sweetened biscuits with a minimum of 30% fruit pomace. However, for people with carbohydrate metabolism disorders or those limiting digestible carbohydrates in their diet, it seems reasonable to choose a product additionally based on erythritol.

### Glycaemic response after ingestion of cookies with fruit pomace

**Figure 3:**
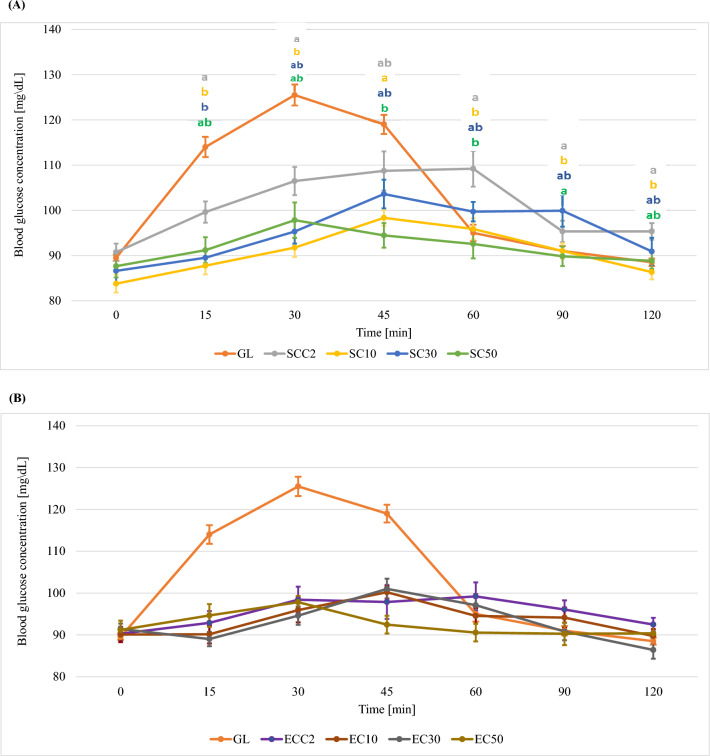
Curves of glycaemic response after consumption of a standard glucose solution and shortbread cookies with different proportions of chokeberry pomace and addition of sucrose (**A**) or erythritol (**B**) (mean value including standard errors—SE). GL—standard glucose solution; S—sucrose; E—erythritol; C—chokeberry pomace; 10, 30, 50—% addition of pomace to flour weight; SCC 1, 2, 3—control cookies sweetened with sucrose; ECC 1, 2, 3—control cookies sweetened with erythritol; a, b—statistically significant differences in glycaemic responses at specific measurement points (the colour of the markings corresponds to a specific cookie variant).

**Figure 4:**
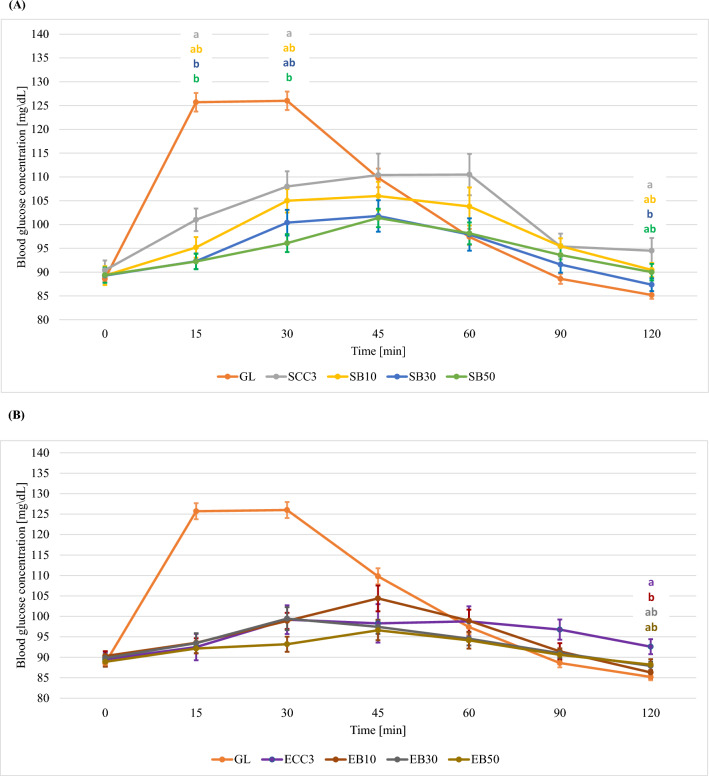
Curves of glycaemic response after consumption of a standard glucose solution and shortbread cookies with different proportions of blackcurrant pomace and addition of sucrose (**A**) or erythritol (**B**) (mean value including standard errors—SE). GL—standard glucose solution; S—sucrose; E—erythritol; B—blackcurrant pomace; 10, 30, 50—% addition of pomace to flour weight; SCC 1, 2, 3—control cookies sweetened with sucrose; ECC 1, 2, 3—control cookies sweetened with erythritol; a, b—statistically significant differences in glycaemic responses at specific measurement points (the colour of the markings corresponds to a specific cookie variant).

**Figure 5:**
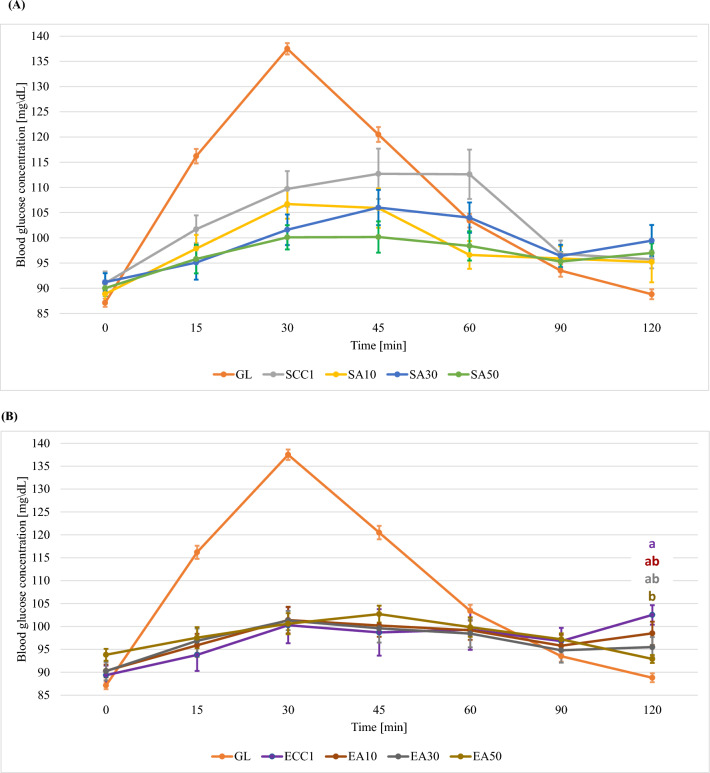
Curves of glycaemic response after consumption of a standard glucose solution and shortbread cookies with different proportions of apple pomace and addition of sucrose (**A**) or erythritol (**B**) (mean value including standard errors—SE). GL—standard glucose solution; S—sucrose; E—erythritol; A—apple pomace; 10, 30, 50—% addition of pomace to flour weight; SCC 1, 2, 3—control cookies sweetened with sucrose; ECC 1, 2, 3—control cookies sweetened with erythritol; a, b—statistically significant differences in glycaemic responses at specific measurement points (the colour of the markings corresponds to a specific cookie variant).

Figures 3–5 show the glycaemic responses after consumption of traditional cookies and cookies with 10%, 30% and 50% added chokeberry, blackcurrant and apple pomace (mean value including standard errors—SE).

#### Shortbread cookies with chokeberry pomace

In the case of chokeberry pomace cookies sweetened with sucrose, it was observed that the addition of pomace significantly influenced the glycaemic response of the study participants 15, 30, 45, 60, 90 and 120 min after consumption (Supplementary Table S1). It was observed that the addition of 10% (SC10) pomace significantly reduced the glycaemic response (15, 30, 60, 90 and 120 min of test) compared to SCC2 cookies (Fig. 3). It can be assumed that this is due to the highest content of dietary fibre and polyphenolic compounds in this variant of cookie. Bajerska et al*.* conducted a study to evaluate the effect of the addition of cherry pomace to muffins on glycaemic response^37^. The authors used a substitution of wheat flour with the addition of cherry pomace. From the analysis of the results, the authors observed that the addition of pomace contributed to a lower glycaemic response 30, 45 and 60 min after consumption, compared to the glycaemic response after consuming muffins without the addition of pomace. In the study by Bajerska et al*.*, maximum glucose concentrations in all muffin variants were recorded in the 30th minute of the test^37^. In our own study, the maximum glucose concentration depended on the addition of chokeberry pomace—at 10 and 30%, the maximum glucose concentration was recorded 45 min into the test, while at 50% addition it was recorded at 30 min (Supplementary Table S1). Maximum glucose levels after ingestion of muffins with 20% and 30% cherry pomace were lower than maximum glucose levels after ingestion of muffins without pomace^37^. The differences in glycaemic responses between the study by Bajerska et al*.*^37^ and our own study are due to the type of products tested (muffins vs. shortbread cookies). Among other things, shortbread cookies are characterized by a higher content of fat, which forms complexes with carbohydrates that require longer enzymatic hydrolysis. The slow digestibility of these complexes reduces the postprandial glycaemic and insulin response in the blood. This leads to a delayed release of glucose and thus a postponement of the maximum glucose concentration over time^38^. The present study also attempted to assess the effect of replacing sucrose with erythritol. Based on a statistical analysis of the results obtained for cookies in which sucrose was replaced by erythritol, no significant effect of the addition of pomace on the glycaemic response at individual measurement points was shown (p ≥ 0.05). However, it was shown that, similar to what was seen for sucrose-sweetened cookies, the maximum blood glucose concentration depended on the percentage of pomace in the recipe. For cookies with 10% and 30% pomace, the highest glycaemic response was observed 45 min into the test. The cookies with the highest proportion of pomace produced the highest glycaemic response 30 min after consumption. The difference in maximum glucose concentration between ECC2 and EC50 cookies was 1.4 mg/dL (Supplementary Table S1, Fig. 3). It was observed that both the type of sweetener used and the percentage of chokeberry pomace in the cookie recipe contributed to lowering the area under the glycaemic curves, but these relationships were not statistically significant. Considering the effect of the sweetener alone, the use of erythritol was shown to significantly reduce the area under the glycaemic curves compared to sucrose (p < 0.001).

#### Shortbread cookies with blackcurrant pomace

Blackcurrant pomace was added to another variant of cookies. It was shown that the effect of addition pomace to sucrose-sweetened cookies significantly affected the glycaemic response only 15, 30 and 120 min after consumption (Supplementary Table S2). Similarly, as in the case of cookies with added chokeberry pomace, SCC3 cookies resulted in an increase in the glycaemic response up to the 60th minute of the test , while for the other variants it increased up to 45th minute. (Supplementary Table S2, Fig. 4). When erythritol was used, the addition of pomace had no significant effect on the blood glucose levels of the study participants. This relationship held true for each measurement point (excluding 120 min of the test—p = 0.0253) (Supplementary Table S2). An attempt was also made to assess the effect of only the addition of the sweetener (without considering the addition of pomace) on blood glucose values. The use of erythritol was shown to significantly reduce the glycaemic response 30, 45 and 60 min into the test (the tested shortbread cookies, sweetened with sucrose, regardless of the addition of pomace, had a significantly higher glycaemic response compared to shortbread cookies sweetened with erythritol). Also in the case of the values of the areas under the glycaemic curves, the replacement of sucrose with erythritol resulted in a significant reduction in their values (regardless of the proportion of pomace in the formulation). (Supplementary Table S2, Fig. 4). Hossain et al*.* conducted a study to evaluate the effect of addition blackcurrant powder and whole wheat flour to shortbread cookies, among others, on carbohydrate digestibility and in vitro glycaemic response^39^. It was observed that all cookie variants showed an increased rate of sugar release during the first 20 min of digestion. In the subsequent digestion process, increasing the addition of blackcurrant powder had a significant effect on reducing the rate of starch degradation in the cookies. Hossain et al*.* did not use an addition level higher than 15%^39^. Analysis of the results of our study showed that the use of pomace is associated with a delay in the onset of maximum blood glucose concentration (45 min into the test). This is most likely related to the fact that blackcurrant and the products resulting from its processing are a source of dietary fibre and antioxidant compounds that may be inhibitors of digestive enzymes^40,41^. Inhibition of enzyme activity may be a consequence of formation of proanthocyanidin and alpha-amylase complexes and starch–phenol interactions that affect its retrogradation^31,42^. A study by Hossain et al*.* showed that as the level of blackcurrant powder addition increased, the areas under the glycaemic curves decreased^39^. In addition, it was observed that the area under the glycaemic curve for cookies with 15% powder addition only differed significantly from that of the control cookies. The present study showed a similar trend—increasing the proportion of pomace resulted in decreasing areas under the glycaemic curves. Considering the addition of pomace at a level similar to that in the study of Hossain et al*.*^39^ (10%), it was observed that the area for this level of addition was not significantly different from the area for SCC3 , or for SB30 and SB50. In our study, a significant difference was shown between SCC3 and SB50 cookies (Supplementary Table S2).

#### Shortbread cookies with apple pomace

The last variant of cookies was products with the addition of apple pomace. It was shown that for both sucrose-sweetened and erythritol-sweetened products, the addition of apple pomace had no significant effect on blood glucose concentrations at specific measurement points. The only exception was for EA50 cookies. In this case, the glucose concentration 120 min into the test was significantly higher (p = 0.0101) than that after consumption of ECC1 cookies (Supplementary Table S3, Fig. 5). Sucrose substitution with erythritol did not significantly affect changes in glucose concentration after ingestion of the products. The only exception was at 45 min into the test (p = 0.0264). Considering the effect of the addition of pomace and the type of sweetener used, it was shown that for sucrose-sweetened cookies, the addition of 50% pomace significantly reduced the area under the glycaemic curve compared to SCC1 (p = 0.0091). For the erythritol-sweetened cookie variants (ECC1, EA10, EA30 and EA50), the addition of pomace had no significant effect on changes in the values of the areas under the glycaemic curves (p = 0.3589). However, a significant reduction in the values of the areas under the glycaemic curves was found when sucrose was replaced by erythritol (without taking into account the addition of pomace) (p = 0.0014). A similar study was carried out by Alongi et al*.*, whose aim was to evaluate the possibility of lowering the glycaemic index of shortcrust pastries by partially replacing wheat flour with apple pomace^10^. During the in vitro digestion process for each cake variant, the amount of glucose released increased rapidly up to 20 min. The researchers observed that the amount of glucose released for the control cookies increased up to 120 min, reaching 120.0 mg/g dry weight. For cakes with 10% and 20% apple pomace, the amount of glucose released also increased after 20 min, but reached lower values of 98.0 and 97.0 mg/g dry weight, respectively^10^. The analysis of the results of this study showed that in the case of cookies sweetened with sucrose, the glucose concentration increased up to 45 min after the start of the test. The slowing of glucose release from ingested products observed in our study may be a consequence of differences in formulation, mainly the different addition of fat (30.3% butter addition in our study vs 8.6% sunflower oil in that of Alongi et al*.*^10^. Our study showed that increasing the addition of apple pomace to shortbread cookies resulted in a significant reduction in the areas under the glycaemic curves. A study by Makarova et al*.*, which assessed the effect of ground apple pomace consumption on the glycaemic response in an oral glucose load test, showed an improvement in glucose metabolism by doubling the glycaemic response 15 and 30 min into the test^43^. Similar observations were made by Schulze et al*.*^44^. They showed that polyphenolic compounds, mainly phloretin, exhibit glucose uptake with the sodium-conjugated glucose transporter-1 (SGLT-1), both in vitro and in vivo. In an oral glucose tolerance test, a reduction in venous blood glucose and plasma insulin levels was observed in study participants.

In the available literature, there is a lack of studies evaluating both the effect of adding different types of food industry waste products and considering sucrose substitutes for the production of pastry and bakery products. Based on the literature review, it is concluded that erythritol may reduce glucose absorption in the small intestine and increase insulin-mediated glucose uptake and metabolism in muscle, which may lead to improved glucose tolerance and alleviation of hyperglycaemic states. In addition, the use of erythritol by both healthy and diabetic individuals has been shown not to increase blood glucose and insulin levels. At the same time, daily consumption of erythritol as a sweetener may be safe for diabetics, not least because it lowers glycated haemoglobin concentrations and may therefore be helpful in the long-term control of postprandial glycaemia^14^.

## Conclusions

The use of waste products from the fruit and vegetable industry in the reformulation of shortbread cookie recipes, involving the partial replacement of wheat flour with pomace and the substitution of sucrose for erythritol, contributed to a reduction in the glycaemic response after their consumption and thus a reduction in their glycaemic index values. Cookies with pomace sweetened with sucrose were classified as products with low and medium GI. (35.5–59.4%), while the erythritol-sweetened cookies were classified in low GI groups (20.7–43.9%). For all variants of sucrose-sweetened cookies, 50% addition of pomace is required to obtain a product with a significantly lower GI compared to control cookies. For erythritol-sweetened cookies, however, no significant effect of adding apple and blackcurrant pomace on GI values was found. Only the use of chokeberry pomace required an increase in its proportion in the recipe to 50%. The results obtained allow us to conclude that it is possible to use waste raw materials to produce functional foods dedicated to people with carbohydrate metabolism disorders.

### Supplementary Information

Supplementary Information 1.

Supplementary Information 2.

Supplementary Information 3.

## Data Availability

Data is provided within the manuscript and supplementary information files.
