# Supplementary material for: Modulation of the glycaemic index value of shortbread cookies by the use of erythritol and fruit pomace
Source: Sci Rep. 2024 Jun 20;14:14215. doi: 10.1038/s41598-024-65108-y (PMC11190288; doi:10.1038/s41598-024-65108-y)
Supplement: Supplementary file 1 — Supplementary Information 1. [file 41598_2024_65108_MOESM1_ESM.docx]

Supplementary Table S1. Blood glucose concentration (mg/dL) of the study participants within 2 hours after the consumption of shortbread cookies with different proportions of chokeberry pomace and addition of sucrose or erythritol

| **Time**  **(min)** | **Sucrose** | | | | | **Erythritol** | | | | | ***p*-S/E** |
| --- | --- | --- | --- | --- | --- | --- | --- | --- | --- | --- | --- |
|  | **% of chocberry pomace addition** | | | | ***p*** | **% of chocberry pomace addition** | | | | ***p*** |  |
|  | **0** | **10** | **30** | **50** |  | **0** | **10** | **30** | **50** |  |  |
|  | 𝑥̅ ± SD | 𝑥̅ ± SD | 𝑥̅ ± SD | 𝑥̅ ± SD |  | 𝑥̅ ± SD | 𝑥̅ ± SD | 𝑥̅ ± SD | 𝑥̅ ± SD |  |  |
| 0 | 90.73±7.43 | 83.73±7.61 | 86.60±8.77 | 87.64±8.31 | 0.0649 | 90.27±6.23 | 90.07±7.02 | 91.40±4.17 | 91.18±7.41 | 0.9598 | 0.0296 |
| 15 | 99.60±9.13a | 87.73±7.48b | 89.50±5.70b | 91.18±9.55ab | 0.0015 | 92.87±10.89 | 90.13±8.51 | 89.00±5.42 | 94.64±9.04 | 0.4796 | 0.7295 |
| 30 | 106.47±12.12a | 91.73±8.08b | 95.30±8.42ab | 97.82±13.04ab | 0.0025 | 98.40±12.09 | 95.93±11.38 | 94.60±7.00 | 97.82±4.98 | 0.8857 | 0.6895 |
| 45 | 108.73±16.71ab | 98.33±6.85a | 103.60±10.05ab | 94.45±9.03b | 0.0225 | 97.87±15.69 | 100.20±5.82 | 101.00±7.69 | 92.45±7.08 | 0.1970 | 0.1342 |
| 60 | 109.20±15.36a | 95.87±9.10b | 99.70±6.83ab | 92.55±10.56b | 0.0031 | 99.20±13.00 | 94.53±7.20 | 97.10±8.49 | 90.55±7.02 | 0.1238 | 0.0885 |
| 90 | 95.33±9.18a | 91.00±6.61b | 99.90±11.18ab | 89.82±7.15a | 0.0301 | 96.07±8.47 | 94.13±9.45 | 90.80±6.58 | 90.27±9.03 | 0.6268 | 0.7740 |
| 120 | 95.33±7.09a | 86.33±6.20b | 90.90±953ab | 88.82±5.88ab | 0.0059 | 92.47±6.29 | 89.73±6.40 | 86.40±6.66 | 90.36±7.49 | 0.1233 | 0.8313 |
| Area under  the curve (j^2^) | 1220.23±159.13 | 913.90±134.75 | 875.67±146.92 | 736.97±160.25 | 0.0664 | 817.47±192.86 | 534.90±112.25 | 474.79±189.44 | 448.59±181.43 | 0.1938 | <0.001 |

𝑥̅ - mean vale; SD - standard deviation; a, b – statistically significant differences in glycaemic responses after consumption of shortbread cookies with different proportions of chokeberry pomace and addition of sucrose or erythritol; *p* - impact of the addition of chokeberry pomace on the glycaemic response or area under the curve depending on the type of sweetener; *p*-S/E – *p*- impact of the addition of sucrose or erythritol on the glycaemic response or the area under the curve.
