# Supplementary material for: Modulation of the glycaemic index value of shortbread cookies by the use of erythritol and fruit pomace
Source: Sci Rep. 2024 Jun 20;14:14215. doi: 10.1038/s41598-024-65108-y (PMC11190288; doi:10.1038/s41598-024-65108-y)
Supplement: Supplementary file 2 — Supplementary Information 2. [file 41598_2024_65108_MOESM2_ESM.docx]

Supplementary Table S2. Blood glucose concentration (mg/dL) of the study participants within 2 hours after the consumption of shortbread cookies with different proportions of blackcurrant pomace and addition of sucrose or erythritol

| **Time**  **(min)** | **Sucrose** | | | | | **Erythritol** | | | | | ***p*-S/E** |
| --- | --- | --- | --- | --- | --- | --- | --- | --- | --- | --- | --- |
|  | **% of blackcurrant pomace addition** | | | | ***p*** | **% of blackcurrant pomace addition** | | | | ***p*** |  |
|  | **0** | **10** | **30** | **50** |  | **0** | **10** | **30** | **50** |  |  |
|  | 𝑥̅ ± SD | 𝑥̅ ± SD | 𝑥̅ ± SD | 𝑥̅ ± SD |  | 𝑥̅ ± SD | 𝑥̅ ± SD | 𝑥̅ ± SD | 𝑥̅ ± SD |  |  |
| 0 | 90.39±7.44 | 89.27±6.59 | 89.27±5.04 | 89.38±5.72 | 0.9226 | 89.54±6.35 | 90.27±3.95 | 89.82±3.34 | 88.85±4.26 | 0.8826 | 0.8014 |
| 15 | 101.08±8.58a | 95.18±7.21ab | 92.27±5.42b | 92.23±5.88b | 0.0155 | 92.54±11.62 | 93.55±3.67 | 93.45±8.14 | 92.15±8.69 | 0.9572 | 0.1247 |
| 30 | 108.08±11.56a | 105.00±8.22ab | 100.36±8.82ab | 96.15±6.83b | 0.0076 | 99.23±12.60 | 98.91±6.59 | 99.45±9.30 | 93.23±6.64 | 0.3941 | 0.0156 |
| 45 | 110.39±16.31 | 106.00±10.05 | 101.82±10.93 | 101.38±7.02 | 0.2161 | 98.31±16.88 | 104.45±10.45 | 97.45±5.80 | 96.62±8.76 | 0.3511 | 0.0151 |
| 60 | 110.54±15.67 | 103.82±13.33 | 97.91±11.37 | 98.15±8.19 | 0.1023 | 98.85±13.19 | 98.95±8.91 | 94.55±5.43 | 94.15±7.30 | 0.6057 | 0.0155 |
| 90 | 95.39±9.61 | 95.46±5.50 | 91.64±5.59 | 93.62±7.87 | 0.6349 | 96.77±8.93 | 91.45±6.62 | 91.00±3.87 | 90.62±5.71 | 0.1067 | 0.3135 |
| 120 | 94.46±6.24a | 90.46±4.84ab | 87.36±4.50b | 90.00±6.28ab | 0.0202 | 92.62±6.63a | 86.27±4.80b | 87.91±3.51ab | 88.23±4.87ab | 0.0253 | 0.1319 |
| Area under  the curve (j^2^) | 1368.19±174.11a | 1043.46±103.72ab | 685.77±416.22b | 648.84±108.58b | 0.0014 | 960.46±537.32a | 782.84±108.39ab | 551.32±85.57ab | 502.15±95.62b | 0.0108 | 0.0285 |

𝑥̅ - mean value; SD - standard deviation; a, b – statistically significant differences in glycaemic responses after consumption of shortbread cookies with different proportions of blackcurrant pomace and addition of sucrose or erythritol; *p* - impact of the addition of blackcurrant pomace on the glycaemic response or area under the curve depending on the type of sweetener; *p*-S/E – *p*- impact of the addition of sucrose or erythritol on the glycaemic response or the area under the curve.
