# Supplementary material for: Modulation of the glycaemic index value of shortbread cookies by the use of erythritol and fruit pomace
Source: Sci Rep. 2024 Jun 20;14:14215. doi: 10.1038/s41598-024-65108-y (PMC11190288; doi:10.1038/s41598-024-65108-y)
Supplement: Supplementary file 3 — Supplementary Information 3. [file 41598_2024_65108_MOESM3_ESM.docx]

Supplementary Table S3. Blood glucose concentration (mg/dL) of the study participants within 2 hours after the consumption of shortbread cookies with different proportions of apple pomace and addition of sucrose or erythritol

| **Time**  **(min)** | **Sucrose** | | | | | **Erythritol** | | | | | ***p*-S/E** |
| --- | --- | --- | --- | --- | --- | --- | --- | --- | --- | --- | --- |
|  | **% of apple pomace addition** | | | | ***p*** | **% of apple pomace addition** | | | | ***p*** |  |
|  | **0** | **10** | **30** | **50** |  | **0** | **10** | **30** | **50** |  |  |
|  | 𝑥̅ ± SD | 𝑥̅ ± SD | 𝑥̅ ± SD | 𝑥̅ ± SD |  | 𝑥̅ ± SD | 𝑥̅ ± SD | 𝑥̅ ± SD | 𝑥̅ ± SD |  |  |
| 0 | 91.00±7.78 | 88.86±6.50 | 91.24±5.92 | 90.00±4.96 | 0.8086 | 89.36±6.93 | 90.27±4.60 | 90.25±6.73 | 93.82±4.38 | 0.3243 | 0.6374 |
| 15 | 101.73±9.17 | 97.86±9.05 | 95.11±11.29 | 95.80±9.46 | 0.3623 | 93.82±11.65 | 95.89±8.37 | 96.87±8.96 | 97.55±7.72 | 0.6947 | 0.4523 |
| 30 | 109.73±11.73 | 106.69±9.49 | 101.58±10.06 | 100.11±7.92 | 0.1201 | 100.27±13.10 | 101.38±9.74 | 101.31±6.88 | 100.58±7.64 | 0.9931 | 0.0860 |
| 45 | 112.73±16.66 | 105.87±12.85 | 105.91±11.73 | 100.16±10.38 | 0.1283 | 98.73±16.83 | 100.20±8.10 | 99.56±10.24 | 102.69±6.16 | 0.8358 | 0.0264 |
| 60 | 112.64±16.22 | 96.56±9.13 | 104.00±9.93 | 98.36±9.69 | 0.0628 | 99.27±14.36 | 99.18±6.95 | 98.45±10.03 | 99.85±6.89 | 0.9627 | 0.0789 |
| 90 | 96.82±8.83 | 95.89±8.05 | 96.40±7.30 | 95.35±6.30 | 0.9693 | 96.82±9.65 | 95.84±7.96 | 94.80±8.90 | 97.18±4.24 | 0.8960 | 0.9777 |
| 120 | 95.73±5.90 | 95.24±13.41 | 99.44±10.21 | 97.05±6.36 | 0.7276 | 102.53±7.09a | 98.51±8.47ab | 95.49±7.38ab | 92.91±2.85b | 0.0101 | 0.7837 |
| Area under  the curve (j^2^) | 1485.41±217.02a | 1170.66±190.66ab | 1039.61±105.58ab | 853.09±118.04b | 0.0091 | 999.91±102.60 | 913.96±153.44 | 810.27±143.56 | 657.82±100.98 | 0.3589 | 0.0014 |

𝑥̅ - mean value; SD - standard deviation; a, b – statistically significant differences in glycaemic responses after consumption of shortbread cookies with different proportions of apple pomace and addition of sucrose or erythritol; *p* - impact of the addition of apple pomace on the glycaemic response or area under the curve depending on the type of sweetener; *p*-S/E – *p*- impact of the addition of sucrose or erythritol on the glycaemic response or the area under the curve.
